# Development of X-Y Servo Pneumatic-Piezoelectric Hybrid Actuators for Position Control with High Response, Large Stroke and Nanometer Accuracy

**DOI:** 10.3390/s100402675

**Published:** 2010-03-25

**Authors:** Mao-Hsiung Chiang

**Affiliations:** Department of Engineering Science and Ocean Engineering, National Taiwan University, No.1, Sec. 4, Roosevelt Rd., Taipei 10617, Taiwan; E-Mail: mhchiang@ntu.edu.tw; Tel.: +882-2-3366 3730; Fax: +882-2-3366 3730

**Keywords:** pneumatic-piezoelectric hybrid actuator, piezoelectric, pneumatic, position control, decoupling self-organizing fuzzy sliding mode control and dual-input single-output

## Abstract

This study aims to develop a X-Y dual-axial intelligent servo pneumatic-piezoelectric hybrid actuator for position control with high response, large stroke (250 mm, 200 mm) and nanometer accuracy (20 nm). In each axis, the rodless pneumatic actuator serves to position in coarse stroke and the piezoelectric actuator compensates in fine stroke. Thus, the overall control systems of the single axis become a dual-input single-output (DISO) system. Although the rodless pneumatic actuator has relatively larger friction force, it has the advantage of mechanism for multi-axial development. Thus, the X-Y dual-axial positioning system is developed based on the servo pneumatic-piezoelectric hybrid actuator. In addition, the decoupling self-organizing fuzzy sliding mode control is developed as the intelligent control strategies. Finally, the proposed novel intelligent X-Y dual-axial servo pneumatic-piezoelectric hybrid actuators are implemented and verified experimentally.

## 1. Introduction

Due to the demand of industrial automation, such as semi-conductor and flat panel display industries, positioning accuracy toward the nanometer range is required, with the working range of centimeters in more than one axis. Research in this field has increased in recent years. Different actuators, with different control strategies, have been discussed. However, the friction force results in the stick-slip effect, such that nanometer accuracy and a working range of centimeters are difficult to reach at the same time.

Piezoelectric actuators have the advantages of a fast response and high positioning accuracy, and have been applied in many different fields, especially in high precision positioning control. The piezoelectric actuator has been used in rigid disk drives for the motion control of a voice coil motor [1]. Non-linear piezoelectric actuator control was implemented by a learning self-tuning regulator [2]. Non-linear modeling of piezoelectric actuators has been discussed [3]. The path tracking control of the piezoelectric actuator was investigated using feedforward compensated PID control [4] and an adaptive rate-dependent feedforward controller [5]. High speed nano-scale positioning was developed using a piezoelectric tube actuator with active shunt control for a scanning probe microscopy [6]. The hysteresis compensation method of the piezoelectric actuator was proposed using modeling of the hysteresis [7]. However, the maximum strokes of piezoelectric actuators can only reach the range of micrometers. Although piezoelectric actuators can perform excellent responses in the nanometer positioning, the strokes are bounded within about the 100 μm range. Thus, they cannot also satisfy the requirement of the positioning system in terms of both positioning accuracy of nanometer range and a working range of centimeters.

Pneumatic systems have the advantages of a high response, easy maintenance and cleanliness, such that it is suitable for automation systems, semi-conductor and flat panel display (FPD) manufacturing equipments, *etc.* However, the pneumatic non-linearities, such as friction force, compressibility, *etc.*, make the pneumatic servo control much more complicated than electric motor servo control and electro-hydraulic servo control, such that the applications in high positioning accuracy are restricted. Research in the field of pneumatic servo control has been developed since the 1960s. Control algorithms, such as PID control, state-space control and adaptive control of pneumatic servo control system, were developed *via* higher speed microcomputers in the 1980s. However, the robustness and the control accuracy of pneumatic cylinders in past research are still unsatisfactory, due to the high non-linearity and the compressibility of compressed air, such that until now, pneumatic servo control was not widely used. The stick-slip effect, which results mainly from friction force of pneumatic cylinders, makes them unable to keep steady motion in low velocity conditions and limits the positioning accuracy. The influence of stick-slip effect on pneumatic servo control systems has been discussed [8]. The non-linear problems complicate pneumatic servo control so that modern control strategies are essential. Self-tuning adaptive control was used in the position control of pneumatic servo cylinders for adapting control parameters on-line, such that the positioning accuracy of 5 μm in no loading conditions was achieved. The additional velocity feedback is inserted in the position control for compensating the influence of friction force [9]. An adaptive sliding-mode controller for position control and path tracking control of a pneumatic system was investigated and the positioning accuracy still remained in the micrometer range [10].

According to the past research, the pneumatic servo control is much more difficult to achieve high accuracy positioning control than the electrical motor servo control and the electro-hydraulic servo control. Therefore, the author has engaged in developing the pneumatic-piezoelectric hybrid actuator for positioning control since 2000 [11,12]. Due to lower friction force, the pneumatic actuator with a double rod is used firstly in the research to combine with the piezoelectric actuator for achieving 180 mm stroke and 0.1 μm accuracy in one motion axis, which is the minimum resolution of the linear scale used in the research [11,12]. According to this, the possibility of nanometer positioning accuracy of the pneumatic-piezoelectric hybrid actuator for large stroke could be expected. If the pneumatic-piezoelectric hybrid actuator can reach the nanometer positioning accuracy, the proposed concept can also be used in electrical motor-piezoelectric hybrid actuators and electro hydraulic-piezoelectric hybrid actuators.

This study aims to investigate an intelligent X-Y dual-axial servo pneumatic-piezoelectric hybrid actuator for position control with dual-axes, high response, large stroke (250 mm) and nanometer accuracy (20 nm). In each axis, the rodless pneumatic cylinder serves to position in the coarse stroke and the piezoelectric actuator compensates the error in the fine range. Thus, the overall control systems of the single axis becomes a dual-input single-output (DISO) system. Since the rodless pneumatic actuator has a relatively higher friction force than the pneumatic actuator with rods used in [11,12], it is more difficult for servo positioning control; however, it has the advantage of mechanism for multi-axes development. Thus, the X-Y dual-axial positioning system is developed based on the servo pneumatic-piezoelectric actuator. Besides, in order to achieve nanometer positioning accuracy, the coupling interaction between the pneumatic and the piezoelectric actuators, as well as that between the X- and Y-axes, must be considered. Therefore, the intelligent controller based on fuzzy sliding mode controller with self-organizing modifier and decoupling ability is proposed for the DISO system of single axis and the X-Y dual-axial systems.

## 2. X-Y Dual-axial Pneumatic-piezoelectric Hybrid Actuator for Positioning Control

**Figure 1.**
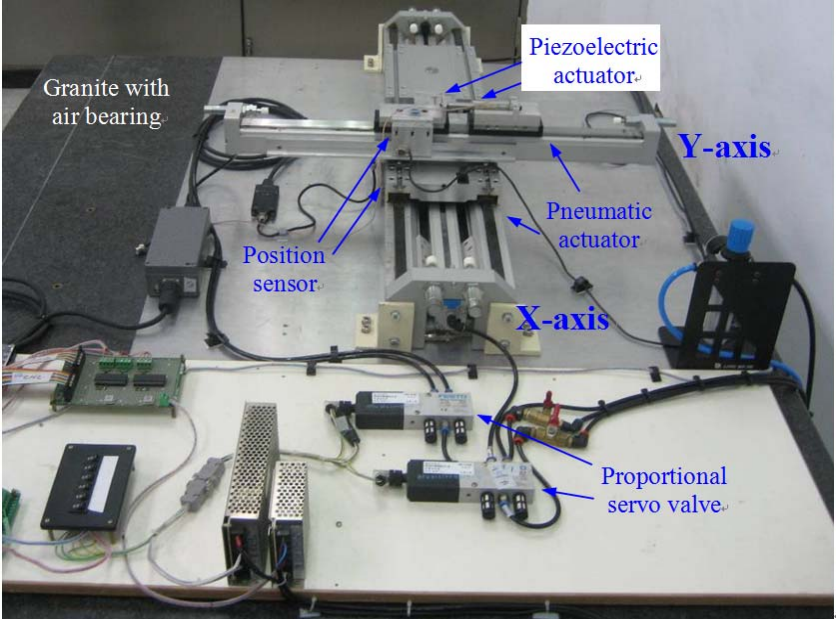
X-Y dual-axial pneumatic-piezoelectric hybrid actuator for position control.

**Figure 2.**
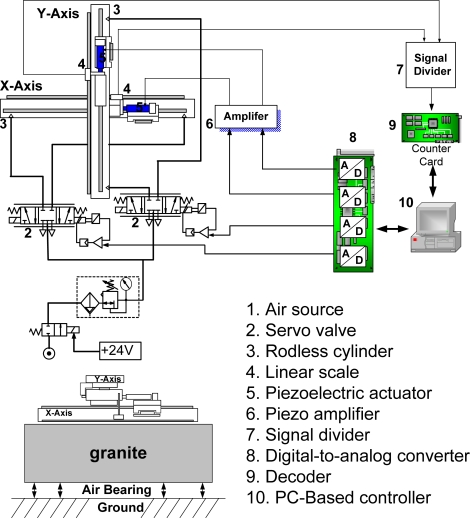
Layout of the X-Y dual-axial pneumatic-piezoelectric hybrid actuator.

Figure 1 photographically illustrates the developed test rig of the novel X-Y pneumatic-piezoelectric hybrid actuator. Figure 2 describes the test rig layout of the novel X-Y pneumatic-piezoelectric hybrid positioning systems. The specifications of the main components are listed in Table 1. Each axis contains a servo pneumatic system and a piezoelectric servo system. Each axis of the pneumatic servo system mainly consists of a rodless pneumatic actuator and a proportional servo valve. In order to achieve the positioning accuracy in the nanometer range, a piezoelectric actuator is mounted in cascade on the piston of the pneumatic actuator *via* an adaptor. Hence, the pneumatic cylinder and the piezoelectric actuator drive the loading mass simultaneously. In addition, the linear encoders with the resolution of 20 nm are equipped for measuring the position of the loading mass, that is, the sum displacement of the pneumatic actuator and the piezoelectric actuator. The measuring signals of the linear encoder are fed back to the PC-Based controller *via* the signal divider and the encoder. The control signals of the pneumatic actuators and the piezoelectric actuators are given from the PC-Based controller *via* the computation of the decoupling intelligent control algorithm with the sampling time of 1 ms *via* digital-to-analog (D/A) converters and enlarged by servo amplifiers and piezoelectric amplifiers, respectively.

**Figure 3.**
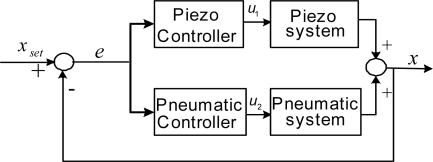
Block diagram of dual-input single-output system for the single axis.

Therefore, the overall control systems of the single axis consist of dual inputs and a single output. Figure 3 illustrates the complex block diagram of the overall control system of the single axis. The servo pneumatic cylinder serves to position in coarse range with high speed and large stroke; the piezoelectric actuator positions in fine stroke for compensating the influence of friction force. The position control of the two control systems, namely pneumatic servo system and piezoelectric servo system, performs in parallel for achieving large stroke, high response and high positioning accuracy simultaneously. The X-Y positioning system is implemented through the combination of the two single axes, and the influence between the motions of the two axes can be considered in the controller design by a decoupling compensator.

## 3. Controller Design

**Figure 4.**
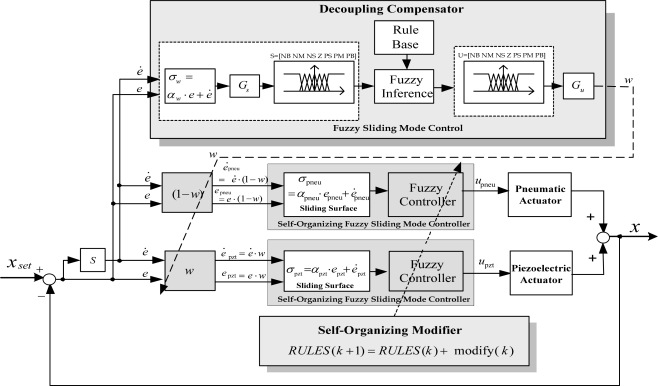
Decoupling self-organizing fuzzy sliding-mode control for single axis.

In this study, decoupling self-organizing fuzzy sliding mode control (DSOFSMC) is developed for solving the DISO control problem and decreasing the fuzzy rule numbers, as well as on-line self-organizing the fuzzy rules. The DSOFSMC contains the self-organizing fuzzy sliding mode control (SOFSMC) and the decoupling compensator. Figure 4 schematically depicts the control block diagram of the DSOFSMC for single axis.

### 3.1. Self-Organizing Fuzzy Sliding Mode Control

Define the position control error vector **e** of the state vector **x** = [*x*, *ẋ*]*^T^* as

(1) $$e=x_{d}-x={\left[e,\dot{e}\right]}^{T}={\left[x_{d}-x,{\dot{x}}_{d}-\dot{x}\right]}^{T}$$

where *x_d_* and *ẋ_d_* indicate the desired output and the desired output change rate; *e* and *ė* are position error and error rate. The fuzzy sliding surface is described as

(2) $$\sigma =\left(\dot{e}+\alpha \;\;e\;\right)=\mathrm{ZERO}$$

where *α* is a positive constant. The fuzzy sliding surface *σ* = *ZERO* is a straight line with slope *α* in the phase plane. The sliding surface can be divided into seven sections by the membership function set of *M* (*σ̃*) = {*NB, NM, NS, ZR, PS, PM, PB*}. The membership function set for the control input *u* is defined as *M* (*ũ*) = {*NB, NM, NS, ZR, PS, PM, PB*}. Therefore, the fuzzy sliding mode control (FSMC) can reduce the fuzzy rules into seven rules *via* the fuzzy sliding surface. The Mamdani method is used in the fuzzy inference and the centroid method is used for defuzzification.

The self-organizing modifier of the SOFSMC is designed by self-organizing fuzzy control theory for on-line adapting the fuzzy rules according to the variations of working points and external disturbance. The detailed derivation of the self-organizing modifier has been shown in [13]. The *i*^th^ rule *u_i_* can be modified by the modifying value Δ*u* through an exciting intensity *w_i_*

(3) $$\begin{array}{l} u_{i}(nT+T)=u_{i}(nT)+w_{i}\cdot \Delta u\\ \;\;\;\;\;\;\;\;\;\;\;\;\;\;\;\;\;\;\;\;\;\;\;\;\;\;\;=u_{i}(nT)+w_{i}\frac{\gamma_{s}}{M_{s}}\sigma (nT)\;\;\;\;\;\;\;,\;\;\;\;i=1,\ldots 7 \end{array}$$

where the exciting intensity *w_i_* is the grade of membership; *γ_s_* indicates the learning rate of the modifier and *M_s_* is a constant ratio between Δ*u* and *σ*(*nT*).

### 3.2. Decoupling Compensator

In order to solve the structure coupling interference, the decoupling compensators designed by SOFSMC are merged. The coupling interactions in the X-Y positioning systems exist not only between the pneumatic actuator and the piezoelectric actuator in the single axis, but also between the X-axis and the Y-axis in the dual-axial motion. They have to be considered respectively.

#### 3.2.1. Decoupling compensator for single axis

Figure 4 shows the control strategies to solve the coupling problem in the DISO system of single axis using DSOFSMC. The decoupling compensator is designed by fuzzy sliding mode control (FSMC).The output of the decoupling controller is considered as weighting function *w*, (0≦ *w* ≦1), for the two subsystems in the DISO system, as described in Equations (4) and (5).

(4) $$\left\{ \begin{array}{l} e_{\mathrm{pneu}}=e\cdot (1-w)\\ {\dot{e}}_{\mathrm{pneu}}=\dot{e}\cdot (1-w) \end{array}\right.$$

(5) $$\left\{ \begin{array}{l} e_{\mathrm{PZT}}=e\cdot w\\ {\dot{e}}_{\mathrm{PZT}}=\dot{e}\cdot w \end{array}\right.$$

where *e_pneu_* and *e_pzt_* are control errors for pneumatic and piezoelectric actuators, respectively.

The overall system of the single axis contains the pneumatic controller, the piezoelectric controller and the decoupling compensator. The pneumatic controller and the piezoelectric controller are designed using SOFSMC; the decoupling compensator is designed by fuzzy sliding mode control (FSMC).

#### 3.2.2. X-Y decoupling compensator

**Figure 5.**
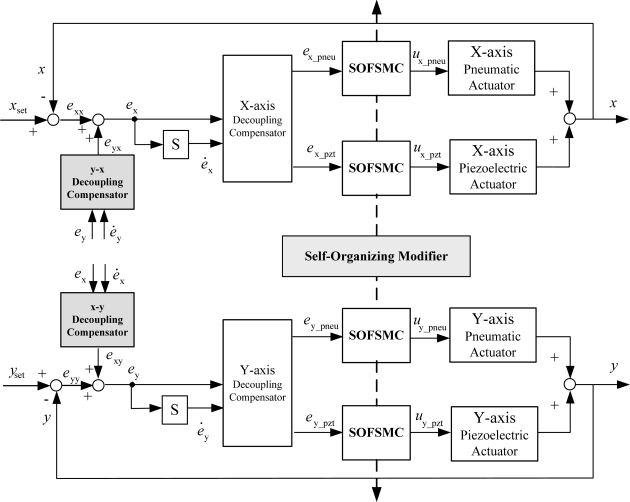
Block diagram of the overall X-Y pneumatic-piezoelectric hybrid positioning system.

Due to the interactions between the motion of the X-axis and Y-axis, the decoupling compensator for the X-Y axes is developed. Figure 5 shows the block diagram of the overall X-Y axes positioning control system. The controllers of the two single axes are designed separately. The coupling influences can be considered by the equivalent control error as

(6) $$\left\{ \begin{array}{l} e_{x}=e_{xx}+e_{yx}\\ e_{y}=e_{yy}+e_{xy} \end{array}\right.$$

where *e_x_* and *e_y_* are resultant position errors of the X- and Y-axis, respectively; *e_xy_* is the equivalent error of the Y-axis caused by the X-axis; *e_yx_* is that of the X-axis caused by the Y-axis. *e_xx_* and *e_yy_* are the feedback control error of the X- and Y-axis. *e_xy_* and *e_yx_* are calculated from the Y-X decoupling compensator that is designed using FSMC.

### 3.3. Controller Design

As shown in Figure 5, the overall control system of the X-Y pneumatic-piezoelectric hybrid positioning system contains the X-axial pneumatic controllers, the X-axial piezoelectric controller, the X-axial decoupling compensator, the y-x decoupling compensator, the Y-axial pneumatic controller, the Y-axial piezoelectric controller, the Y-axial decoupling compensator, and the x-y decoupling compensator. The decoupling self-organizing fuzzy sliding mode controller is used in the X-axial pneumatic controller, the X-axial piezoelectric controller, the Y-axial pneumatic controller and the Y-axial piezoelectric controller. The X-axial decoupling compensator, the y-x decoupling compensator, the Y-axial decoupling compensator and the x-y decoupling compensator are design using fuzzy sliding mode control. The control designed parameters of the controllers are summarized in Table 2, where *G*_s_ is the normalized factor and *G*_u_ the defuzzification factor; *α* is the slope of fuzzy sliding surface; *γ*/ *M* is the learning rate ;*T* is the membership function.

## 4. Experiments

For ensuring feasibility of the design of the mechanism and the controllers for the novel X-Y pneumatic-piezoelectric hybrid positioning control system, the experiments for different strokes for pneumatic actuator, piezoelectric actuator, pneumatic-piezoelectric hybrid actuators for single axis and dual axes are implemented. In addition, the bi-directional test and the robustness test are also performed.

### 4.1. Position Control of Pneumatic Actuator

**Figure 6.**
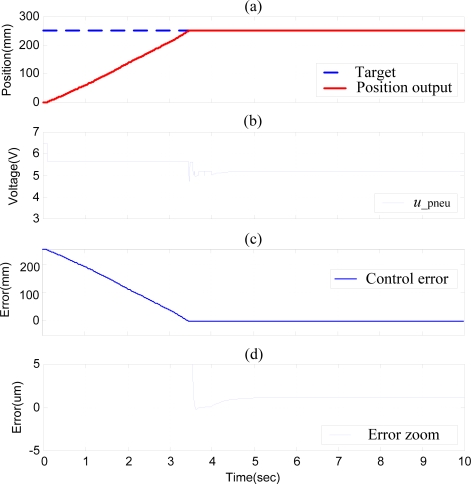
Experimental results of position control of the X-axial pneumatic actuator with stroke of 250 mm by SOFSMC: (a) position control response, (b) control signal, (c) control error, (d) error zoom.

The pneumatic actuator controlled by the SOFSMC is investigated firstly to identify the position control error range. Figure 6 shows the experimental results of the position control of the X-axial pneumatic actuator with stroke of 250 mm. The settling time is about 3.53 s and the steady state control error can achieve about 1 μm.

### 4.2. Position Control of Piezoelectric Actuator

**Figure 7.**
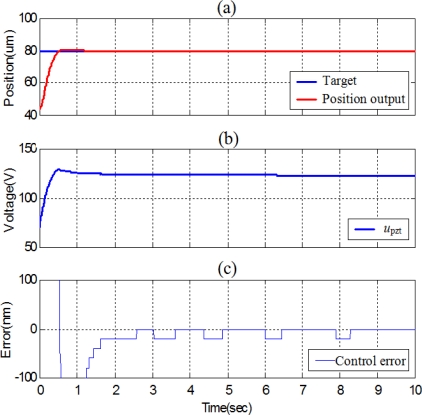
Experimental results of position control of the X-axial piezoelectric actuator with stroke of 80 μm by SOFSMC: (a) position control response, (b) control signal, (c) control error.

The experimental position control performance of the piezoelectric actuator controlled by the SOFSMC is shown in Figure 7. Because the maximum stroke of the piezoelectric actuator used in the study is 100 μm with the maximum driving voltage of 150 V, the experimental results of the position control of the piezoelectric actuator with stroke of 80 μm is performed. In order to make the piezoelectric actuator able to compensate the position on both sides, the control signal of 4.67 V, which is amplified to 70 V by the piezoelectric amplifier, is pre-given. The rise time, the settling time and the steady state control error can achieve 0.635 s, 1.255 s and 20 nm, respectively.

### 4.3. Position Control of the Pneumatic-Piezoelectric Hybrid Actuator for Single Axis

The position control of the pneumatic-piezoelectric hybrid actuator for single axis is implemented to confirm if the required large stroke and nanometer positioning accuracy can be realized. In addition, the position control performances with and without decoupling compensator of single axis are compared. Furthermore, the double step position control is realized to verify the proposed hybrid actuator in bi-directional motions.

**Figure 8.**
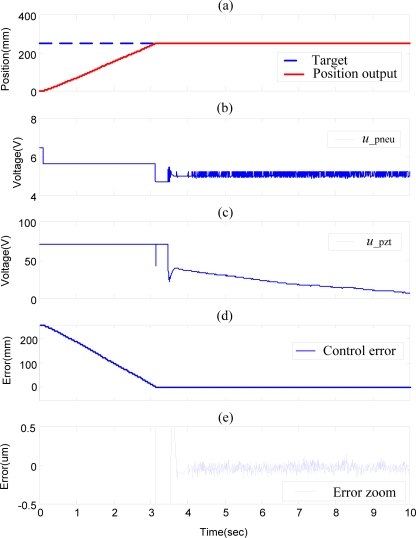
Experimental results of position control of the pneumatic-piezoelectric hybrid actuator for single axis with stroke of 250 mm by SOFSMC (without decoupling compensator): (a) position control response, (b) pneumatic control signal, (c) piezoelectric control signal, (d) position control error, (e) error zoom.

Figure 8 shows the experimental results of position control of pneumatic-piezoelectric hybrid actuator for single axis with a stroke of 250 mm by SOFSMC, *i.e.*, without decoupling compensator. The pneumatic actuator firstly compensates the position control error to reach micrometer range, and subsequently the piezoelectric actuator starts to compensate the control error in the fine range. However, the pneumatic actuator and the piezoelectric actuator interfere with each other in the fine range, such that the control error chatters seriously over 0.1 μm, as shown in Figure 8d.

**Figure 9.**
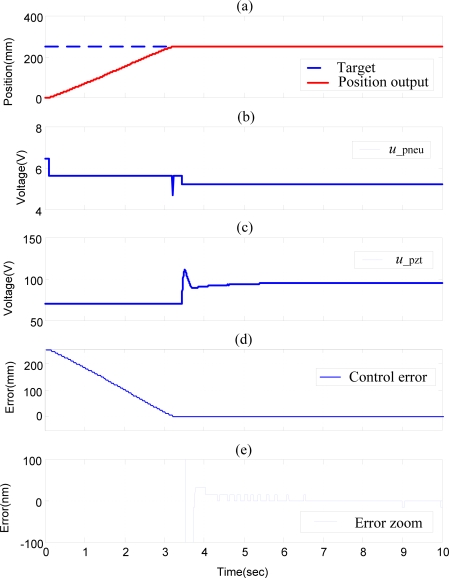
Experimental results of position control of pneumatic-piezoelectric hybrid actuator for single axis with stroke of 250 mm by DSOFSMC (with decoupling compensator): (a) position control response, (b) pneumatic control signal, (c) piezoelectric control signal, (d) position control error, (e) error zoom.

Therefore, the decoupling compensator of the single axis is essential for tackling the coupling influence. The position control of the pneumatic-piezoelectric hybrid actuator for single axis with stroke of 250 mm by DSOFSMC, *i.e.*, with decoupling compensator of single axis, is shown in Figure 9. The steady state control error in Figure 9(d) clarifies that the decoupling compensator can effectively solve the coupling problem and can make the steady state error reach 20 nm at 3.695 s.

**Figure 10.**
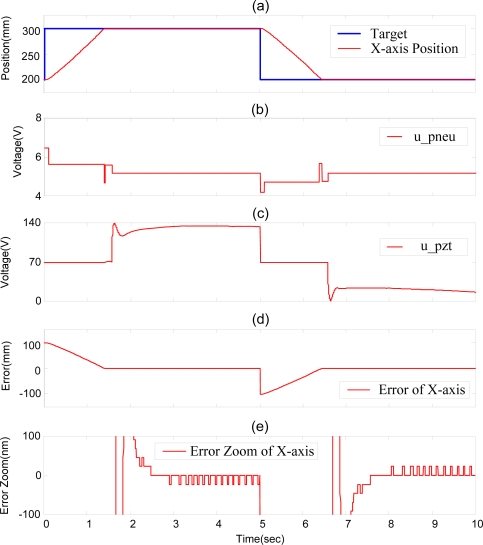
Experimental results of bi-directional position control of the pneumatic-piezoelectric hybrid actuator for single axis with stroke of 100 mm by DSOFSMC: (a) position control response, (b) pneumatic control signal, (c) piezoelectric control signal, (d) position control error, (e) error zoom.

Moreover, Figure 10 shows the experimental results of bi-directional position control of pneumatic-piezoelectric hybrid actuator for single axis with stroke of 100 mm by DSOFSMC. The proposed novel hybrid actuator can achieve the positioning accuracy of 20 nm in both forward and backward directions.

### 4.4. Robustness Test of the Pneumatic-Piezoelectric Hybrid Actuator for Single Axis

**Figure 11.**
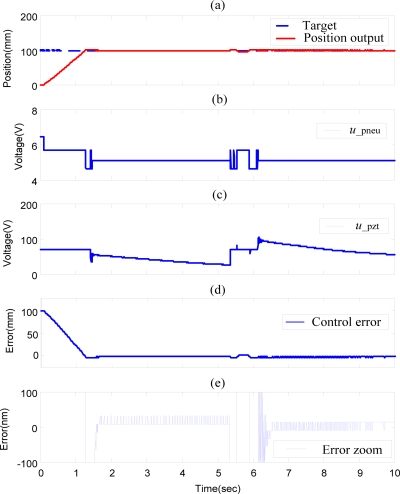
Robustness test of the position control of pneumatic-piezoelectric hybrid actuator for single axis by DSOFSMC: (a) position control response, (b) pneumatic control signal, (c) piezoelectric control signal, (d) position control error, (e) error zoom.

In order to investigate the proposed pneumatic-piezoelectric hybrid actuator against disturbance, the robustness test was performed, as shown in Figure 11. The initial inertia mass, which contains the piston and mechanism of pneumatic cylinder, as shown in Figure 1, is about 4.2 kg for the X-axis and 2.5 kg for the Y-axis. A disturbance loading mass of 5 kg is inserted during the steady state such that the control error suddenly increases over the maximum stroke of the piezoelectric actuator ± 50 μm; meanwhile, the pneumatic actuator works immediately to compensate the error and then the piezoelectric actuator makes the control error return quickly to 20 nm. Thus, the DSOFSMC that combines the decoupling compensator with the SOFSMC is effectively for the pneumatic-piezoelectric hybrid actuator for the variation of disturbance force and mass.

### 4.5. Position Control of the XY Dual-Axial Pneumatic-Piezoelectric Hybrid Actuators

**Figure 12.**
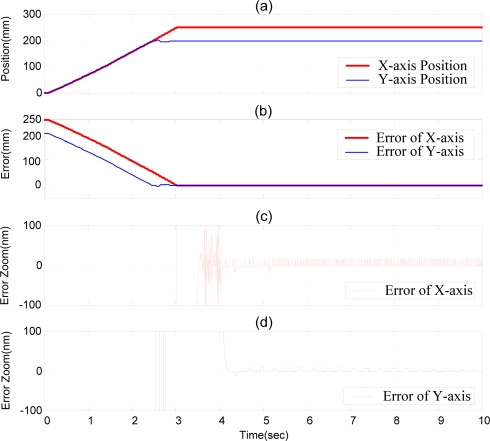
Experimental results of the positioning control of XY dual-axial pneumatic-piezoelectric hybrid actuators by DSOFSMC without X-Y decoupling compensator: strokes of (X,Y)= (250 mm, 200 mm), (a) position control response of X- and Y-axes. (b) error of X- and Y-axis. (c) error zoom of X-axis. (d) error zoom of Y-axis.

With the purpose of developing the XY dual-axial pneumatic-piezoelectric hybrid actuators for large stroke and nanometer positioning accuracy, the coupling interactions between the motions of the X- and Y-axes have to be considered. Although the X- and Y-axis are theoretically orthogonal, the clearance of the linear bearings and the orthogonal mounting deviation between X- and Y-axis still exist in the practical test rig. The XY-decoupling compensator was used here to compensate the mechanism deviation. For this purpose, two experiments of the XY dual-axial position control with the stroke of (250 mm, 200 mm) for the X- and Y-axis were performed. Figure 12 shows the experimental results of the positioning control of the XY dual-axial pneumatic-piezoelectric hybrid actuators controlled by DSOFSMC without the X-Y decoupling controller. Figure 12a shows the position control response; Figure 12b, c are the positioning error zoom of X- and Y-axis. Without the X-Y decoupling controller, the motions of X- and Y-axis influence obviously on each other, such that the serious oscillations appear before the steady state in both X- and Y-axial responses.

**Figure 13.**
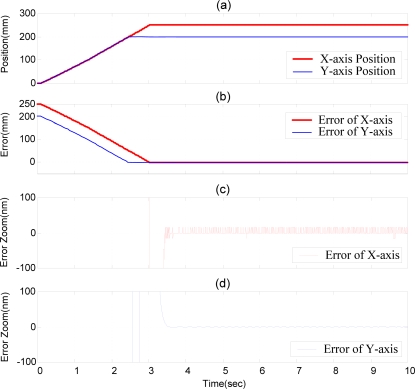
Experimental results of the positioning control of XY dual-axial pneumatic-piezoelectric hybrid actuators by DSOFSMC with X-Y decoupling controller: strokes of (X,Y)= (250 mm, 200 mm), (a) position control response of X- and Y-axes. (b) error of X- and Y-axis. (c) error zoom of X-axis. (d) error zoom of Y-axis.

**Figure 14.**
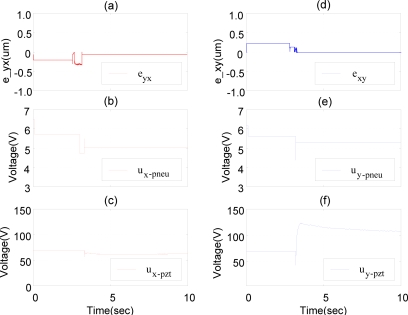
Control signals of the positioning control of XY dual-axial pneumatic-piezoelectric hybrid actuators by DSOFSMC with X-Y decoupling controller: strokes of (X,Y) = (250 mm, 200 mm), (a) equivalent control error *e*_yx_, (b) pneumatic control signal of X-axis, (c) piezoelectric control signal of X-axis, (d) equivalent control error *e*_xy_, (e) pneumatic control signal of Y-axis, (f) piezoelectric control signal of Y-axis.

Figure 13 schematically depicts the experimental results of the positioning control of XY dual-axial pneumatic-piezoelectric hybrid actuator controlled by DSOFSMC with the X-Y decoupling controller. The strokes of the X- and Y-axis are (250 mm, 200 mm). With the X-Y decoupling controller, the dual-axial coupling interactions can be reduced clearly, such that almost no oscillations occur before the steady state in both X- and Y-axis responses, as shown in Figure 13b, c. The X- and Y-axial pneumatic-piezoelectric hybrid actuator can achieve the position accuracy of 20 nm for the strokes of 250 mm and 200 mm. The variations of the equivalent control error *e*_yx_, the pneumatic control signals of X-axis, the piezoelectric control signals of X-axis, the equivalent control error *e*_xy_, the pneumatic control signals of Y-axis, and the piezoelectric control signals of Y-axis are shown in Figure 14.

For comparing the position control responses of X- and Y-axis with and without the X-Y decoupling controller, Table 3 summarizes the rising time and the steady state error of the experiments shown in Figures 12 and 13. In the X-axis response, the steady state error both can reach 20 nm, and the system with X-Y decoupling controller can reduce the oscillations and have better steady state time of 3.425 s. In the Y-axis response, the steady state error also can reach 20 nm. The system with X-Y decoupling controller has better steady state time of 3.553 s.

## 5. Conclusions

This paper proposes a novel large stroke and high precision XY dual-axial pneumatic-piezoelectric hybrid actuators in which the pneumatic servo cylinders serve for high speed, large stroke positioning and the piezoelectric actuators work in fine stroke positioning for each axis. The two controllers work in parallel and the two position outputs are added in cascade. Such complex dual-input single-output systems are solved by the decoupling self-organizing fuzzy sliding mode control both for the pneumatic cylinder positioning control system and the piezoelectric positioning control system and the decoupling compensator of single axis. In addition, for reducing interactions between the motion of the X- and Y-axis, the X-Y decoupling controllers for the X-Y axes are developed and verified.

The experimental results clarify that the servo pneumatic-piezoelectric X-Y hybrid control systems can achieve excellent positioning response and accuracy of 20 nm with high response for maximum stroke of 250 mm and 200 mm for the X- and Y-axis. Besides, the 20 nm positioning accuracy is limited by the resolution of the linear scales. If the resolution of the linear scale and the counter speed of the decoder could be improved, the positioning accuracy and the transient response could be improved further.

## Figures and Tables

**Table 1.** Specifications of the main components of the X-Y pneumatic-piezoelectric hybrid actuator.

Component	Specification
Pneumatic actuator	X-axis: Piston diameter: φ25 mm Stroke: 600 mm Y-axis: Piston diameter: φ25 mm Stroke: 500 mm
Piezoelectric actuator	Stack type Stroke: 100 μm Driving voltage: 0∼150 V
Piezoelectric amplifier	Input : 0∼5V Output: 0∼150 V
Proportional servo valve	5/3 directional flow control valve Driving voltage:0∼10 V Rated volume flow: 700 L/min. Max. hysteresis: 0.4% –3 dB Bandwidth: 125 Hz for 100% stroke
Linear encoder	Resolution: 20 nm
Data acquisition card	12 bits D/A (digital-to-analog); 16 bits DIO (digital input/digital output)
Counter card	3 × 24-bits counters 1.0 MHz input rate
PC-Based Controller	Industrial PC Pentium 4 CPU

**Table 2.** Parameters of controllers.

(a) Pneumatic controller	
*α*_pneu_ = 50	*G*_s_ = 0.1
*G*_u_ = 0.8	*γ*_V_ / *M*_V_ = 0.001
*T*(*σ*_pneu_) ={−1, −0.67, −0.1, 0, 0.1, 0.67, 1}	
*T*(*u*_pneu_) ={−1 −0.66 −0.33 0 0.33 0.66 1}	

(b) Piezoelectric controller	
*α*_pzt_ = 50	*G*_s_ = 4.8
*G*_u_ = 0.4	*γ*_V_ / *M*_V_ = 0.0007
*T*(*σ*_pzt_) ={−1 −0.25 −0.01 0 0.01 0.25 1}	
*T*(*u*_pzt_) ={−0.5 −0.22 −0.01 0 0.01 0.22 0.5}	

(c) Decoupling compensator - single axis	
*α*_w_ = 4	*G*_s_ = 0.5
*G*_u_ = 0.8	
*T*(*σ*_pzt_) ={−1 −0.4 −0.1 0 0.1 0.4 1}	
*T*(*u*_pzt_) ={−1 −0.6 −0.3 0 0.3 0.6 1}	

(d) Decoupling compensator—XY axis	
*α*_xy_ = 4	*G*_s_ = 0.02
*α*_yx_ = 4	*G*_u_ = 0.04
*T*(*σ*_yx_) ={−0.1 −0.004 0 0.005 0.1}	
*T*(*u*_yx_) ={−0.025 −0.0125 0 0.0125 0.025}	
*T*(*σ*_xy_) ={−0.1 −0.005 0 0.0005 0.1}	
*T*(*u*_xy_) ={−0.05 −0.025 0 0.025 0.05}	

**Table 3.** Comparison of the positioning responses of X- and Y-axial pneumatic-piezoelectric hybrid actuators with and without X-Y decoupling controller.

	X-axis response		Y-axis response	
	without X-Y decoupling controller	with X-Y decoupling controller	without X-Y decoupling controller	with X-Y decoupling controller
Rising time *t_r_*	3.034 s	3.035 s	2.545 s	2.555 s
Steady state error *e_ss_*	20 nm at 4.035 s	20 nm at 3.425 s	20 nm at 4.205 s	20 nm at 3. 553 s
